# Physical demands during 3 × 3 international male and female basketball games are partially impacted by competition phase but not game outcome

**DOI:** 10.5114/biolsport.2023.116012

**Published:** 2022-06-01

**Authors:** Davide Ferioli, Ermanno Rampinini, Daniele Conte, Diego Rucco, Marco Romagnoli, Aaron Scanlan

**Affiliations:** 1 1UCAM Research Center for High Performance Sport, Catholic University of Murcia, Murcia, Spain; 2 Human Performance Laboratory, MAPEI Sport Research Centre, Olgiate Olona, Varese, Italy; 3 Institute of Sport Science and Innovations, Lithuanian Sports University, Kaunas, Lithuania; 4 Independent Researcher; 5 Faculty of Science of Physical Education and Sport, University of Valencia, Valencia, Spain; 6 Human Exercise and Training Laboratory, School of Health, Medical, and Applied Sciences, Central Queensland University, Rockhampton, Queensland, Australia

**Keywords:** 3-on-3 basketball, Time-motion analysis, Activity demands, External load, Match analysis, High-intensity activity, Team sports

## Abstract

Despite the increased popularity and number of international competitions for 3 × 3 basketball, the precise physical demands of this sport are not well described. Therefore, this study aimed to quantify the physical demands of 3 × 3 basketball games according to game outcome and competition phase. Following an observational design, video footage from 27 games were analysed from 104 international 3 × 3 basketball players (n = 52 male and n = 52 female players) belonging to 26 national teams (n = 13 male and n = 13 female teams) during the 2019 FIBA 3 × 3 World Cup. Manual frame-by-frame time-motion analyses were conducted to determine the relative frequency (n · min^−1^) and duration (% of live playing time) for several physical demand variables to make comparisons according to game outcome (win vs. loss) and competition phase (group games vs. finals games). Linear mixed models for repeated measures and effect size analyses revealed non-significant, trivial-small differences in physical demands between games that were won and lost. Regarding competition phase, male players completed more high-intensity activity (sprinting, high-intensity specific movements, and jumping) but spent a greater proportion of playing time jumping and performing recovery activities (standing/ walking) during finals games than group games (P < 0.05, small), while female players performed more low-intensity activity (jogging and low-intensity specific movements) during group games than finals games (P < 0.05, small). These findings indicate that the physical capabilities of male and female 3 × 3 basketball players may not be the determining factor for team success in games and players can mostly maintain activity outputs across phases of tournament play conducted at the highest international standard.

## INTRODUCTION

Although 3 × 3 basketball is a global team sport adapted from the traditional 5 × 5 version of basketball, its unique characteristics likely impose specific demands on players compared to 5 × 5 basketball. In this regard, 3 × 3 basketball games are played on smaller courts (15 m × 11 m vs. 15 m × 28 m), with a reduced number of players competing at any one time (3 vs. 5), and following shorter overall live playing times (10 min vs. 40–48 min) and shot clock durations per possession (12 s vs. 24 s) compared to 5 x 5 basketball games. Accordingly, increased research is emerging exploring the demands of 3 × 3 basketball [1–8] so that team training plans and tactical strategies, as well as player technical skills and physical characteristics can be optimally developed to suit these demands.

Studies quantifying the demands of 3 × 3 basketball games have measured the technical-tactical demands [1–6], internal load [6–8], and external load [7, 8] of junior and adult players across both sexes competing at national and international levels. Existing data indicate 3 × 3 basketball games evoke high internal loads including heart rates of ~165 beats · min^−1^, blood lactate concentrations > 6.0 mmol · L^−1^, and ratings of perceived exertion > 5 arbitrary units (AU) using a modified Borg CR10 scale in male and female junior (under 18 years) and adult international players [6–8]. Regarding external load data during 3 × 3 basketball games, only two studies [7, 8] have quantified physical demands using positioning system and inertial sensor technology.

These studies [7, 8] have shown male and female players competing in the 2016 International Basketball Federation 3 × 3 under-18 World Championships, Senior European, and World Championships cover > 850 m and experience absolute PlayerLoads^TM^ ranging from 116 ± 29 to 133 ± 28 AU and relative PlayerLoads^TM^ ranging from 6.3 ± 1.4 to 6.8 ± 1.5 AU · min^−1^ during games. However, further data indicating the precise movement types and intensities completed by players during 3 × 3 basketball games is needed to provide practitioners with a more thorough understanding of the physical demands faced by players for enhanced translation to practice. In this way, video-based time-motion analysis (TMA) represents a valid [9–12] and reliable [9, 10, 12–15] method to provide detailed insight regarding the movements performed by players not captured with positioning system and inertial sensor technology such as stressful stationary activity (e.g., screens, boxing out opponents during rebounds, holding position on the court) [16]. Consequently, further research quantifying physical demands using approaches not adopted in the previous literature, such as video-based TMA, are needed to understand the complete requirements of players more comprehensively during 3 × 3 basketball games.

When quantifying player physical demands during basketball games, contextual factors should be considered given they may affect the activities performed by players [17]. Two key contextual factors that have received increased research attention when quantifying game demands in basketball players include game outcome (win vs. loss) [1, 17–22] and competition phase (group phase vs. final phase) [6, 7]. Regarding game outcome, previous research has only explored technical-tactical demands during 3 × 3 basketball games reporting winning male and female teams competing in the 2019 European Basketball Cup performed significantly better than losing teams in various metrics including scoring more efficiently (i.e., points per possession), securing more rebounds, committing fewer turnovers, and committing less fouls [3]. However, no data of this kind on physical demands according to game outcome are yet available for 3 × 3 basketball players. Regarding competition phase, research quantifying the technical-tactical demands of male and female players competing in 3 × 3 basketball games during the 2019 European Basketball Cup showed significantly greater scoring efficiency (i.e., points per possession) and less blocks were completed during group games compared to finals games [3]. In turn, research examining male and female players during the 2019 Australian National Basketball League 3 × 3 Pro Hustle demonstrated significantly less shots (semi-finals), more offensive rebounds (quarter-finals), and less steals (quarter-finals and semi-finals) were completed during finals games compared to group games. Further to technical-tactical demands, research exploring the internal responses of male and female players during 3 × 3 games found significantly higher ratings of perceived exertion (semi-finals and finals) and peak heart rates (semi-finals) during finals games compared to group games during the 2019 Australian National Basketball League 3 × 3 Pro Hustle. Nevertheless, no data exist directly comparing the physical demands of 3 × 3 basketball games between finals and group phases, with data only reported for individual games in male and female players across under-18 and senior international tournaments [7].

Due to the limited findings reported in the literature regarding the physical demands associated with 3 × 3 basketball games and considering no research has directly explored the impact of game outcome and competition phase on these demands, research on this topic is essential. Such data will assist basketball coaches and high-performance staff in identifying the most influential physical factors contributing to in-game success for the development of training plans that optimally prepare players for 3 × 3 basketball competition. Therefore, the aim of this study was to quantify the physical demands of 3 × 3 basketball games according to game outcome (win vs. loss) and competition phase (group games vs. finals games).

## MATERIALS AND METHODS

### Subjects

Data were collected from 104 international 3 × 3 basketball players (n = 52 male and n = 52 female players) belonging to 26 national teams (n = 13 male and n = 13 female teams), with each team comprised of four players. The study was approved by the Independent Institutional Review Board of MAPEI Sport Research Centre in accordance with the Helsinki Declaration (2013).

### Design

An observational study design was used to assess the physical demands experienced during games in male and female players competing at the 2019 FIBA 3 × 3 World Cup according to game outcome (win vs. loss) and competition phase (group phase vs. finals phase). This competition featured 20 male and 20 female teams, that were divided into 4 groups of 5 teams, with teams seeded automatically based on the 3 × 3 FIBA world rankings. Separately in the male and female competitions, the top two teams in each group following the group phase progressed to quarter-final games. The winner of each quarter-final game progressed to the semi-final games, with winners of semi-final games progressing to the final. The games were played in accordance with the FIBA 3 × 3 basketball rules (https://fiba3x3.com/docs/fiba-3x3-basketball-rules-full-version.pdf). Video footage for each game was publicly accessible online via the tournament website (https://www.fiba.basketball/3x3WC/2019) or Youtube (https://www.youtube.com/c/FIBA3x3/playlists). A total of 27 official games out of the 96 games disputed during the 2019 FIBA 3 × 3 World Cup were included in final analyses. Specifically, all the games involving two teams not qualifying for the finals phase were excluded (n = 24 games) to reflect a similar distribution of games between competition phases for analyses. Then, games with more than 3% of total playing time not available for technical reasons (e.g., video interruption, commercial break, action replays) were excluded (n = 45 games). Specifically, 16 games during the group phase (male games, n = 8; female games, n = 8) out of 80 total games during the group phase and 11 games during the finals phase (male games, n = 5 [final for first and second place, n = 1; semi-final, n = 1 and quarter-finals, n = 3]; female games, n = 6 [final for first and second place, n = 1; final for third and fourth place, n = 1; semi-finals, n = 2 and quarter-finals, n = 2]) out of 16 total games during the finals phase were analysed, thus resulting in a total of 216 (male games, n = 104 [group phase, n = 40; finals phase, n = 64] and female games, n = 112 [group phase, n = 48; finals phase, n = 64]) individual game samples being analysed.

### Time-motion analysis

A manual frame-by-frame approach was used to determine player physical demands during games using established software (SICS VideoMatch Basket, version 5.0.5). As previously described [9, 14, 15], physical demands were quantified by identifying movements into one of eight categories as follows: (i) stand/walk: movement of no greater intensity than walking without any distinction between standing still and walking or between different intensities of walking; (ii) jog: forwards or backwards movement at an intensity greater than walking but without urgency; (iii) run: forwards or backwards movement at an intensity greater than jogging and a moderate degree of urgency but which did not approach an intense level of movement; (iv) sprint: forwards or backwards movement at a high intensity, characterised by effort and purpose at or close to maximum; (v) low-: (vi) moderate-: (vii) high-specific movements (SM): movements differing from ordinary standing, walking or running performed respectively at low intensity without urgency, at medium intensity with a moderate degree of urgency, and at high intensity with urgency; and (viii) jump: the time from the initiation of the jumping action to the completion of landing. SMs mainly included the stance position, shuffling, rolling, reversing, screening, and cross-over running activities [23]. Activities in each of these eight categories were then grouped more broadly according to their relative intensity as: (i) recovery (REC, i.e., standing/walking); (ii) low-intensity activities (LIA, i.e., jogging and low-SM); (iii) medium-intensity activities (MIA, i.e., running and moderate-SM); and (iv) high-intensity activities (HIA, i.e., sprinting, high-SM, and jumping) [9, 14, 15]. The frequency of occurrence and the duration of each activity were determined during live playing time (i.e., when the game clock was running). Activity frequencies were calculated as the total number of events (n) performed and normalised according to playing time (n · min^−1^) for each player to account for the varying exposures and substitution times across players. Activity durations were determined as a percentage (%) of the playing time for each player to account for the varying exposures and substitution times across players. The analysis was carried out by a single experienced video analyst. Intra-tester reliability was determined by having the observer analyse the relative frequency (n · min^−1^) and duration (s) of activities during an entire 2019 FIBA 3 × 3 World Cup 3 × 3 game for all players (n = 8) on two occasions separated by 7 days to minimise mental recall of video footage. The reliability of this approach through calculation of the intraclass correlation coefficient (ICC) and coefficient of variation (CV) were deemed acceptable and are reported in Table 1.

**TABLE 1:** Intra-tester reliability of video-based time-motion analysis variables reported in this study.

Variable	Movement category	ICC (95%CI)	CV% (95%CI)
Frequency of occurrence	REC	0.97 (0.87–0.99)	5.62 (3.68–11.78)
	LIA	0.98 (0.90–1.00)	3.92 (2.57–8.14)
	MIA	0.99 (0.94–1.00)	4.00 (2.63–8.31)
	HIA	0.99 (0.96–1.00)	2.90 (1.91–6.00)
	Sprint	0.99 (0.97–1.00)	5.14 (3.37–10.75)
	High-SM	0.97 (0.84–0.99)	6.60 (4.31–13.88)
	Jumps	1.00 (1.00–1.00)	0.00 (0.00–0.00)

Duration	REC	1.00 (0.98–1.00)	3.17 (2.09–6.56)
	LIA	0.99 (0.94–1.00)	3.52 (2.31–7.30)
	MIA	0.95 (0.76–0.99)	8.68 (5.66–18.46)
	HIA	1.00 (0.92–1.00)	2.04 (1.34–4.20)
	Sprint	0.98 (0.94–1.00)	8.41 (5.49–17.87)
	High-SM	0.98 (0.91–1.00)	5.10 (3.35–10.66)
	Jumps	0.99 (0.96–1.00)	5.09 (3.34–10.63)

*Abbreviations*: ICC, intraclass correlation coefficient; CI, confidence intervals; CV%, coefficient of variation as a percentage; REC, recovery; LIA, low-intensity activities; MIA, medium-intensity activities; HIA, high-intensity activities; SM, specific movements.

### Statistical analysis

The TMA descriptive results are reported as means ± standard deviations (SD). Linear mixed models were constructed to examine differences in REC, LIA, MIA, HIA, total movements, sprint, high-SM, and jump data according to game outcome and competition phase, accounting for individual repeated measures. Linear mixed models were separately constructed for males and females where game outcome (2 levels: win and loss) and competition phase (2 levels: group phase and finals phase) were used as fixed effects and player as a random effect with a random intercept and fixed slope. All assumptions were met including assessing the normality of the residuals using the Kolmogorov-Smirnov test. Moreover, the magnitude of differences was assessed using effect size (ES) analyses with 95% confidence intervals and interpreted as: < 0.2 = *trivial*; 0.20–0.59 = *small*; 0.60–1.19 = *moderate*; 1.2–1.99 = *large*; and ≥ 2.0 = *very large* [24]. An alpha level of < 0.05 was set *a priori* for statistical significance. All data were analysed using Jamovi software (version 2.0.0.0, 2021).

## RESULTS

Descriptive data and statistical analyses for physical demand variables during male basketball games according to game outcome (i.e., win vs. loss) and competition phase (i.e., group games vs. finals games) are presented in Tables 2 and 3. Regarding comparisons according to game outcome, no significant differences were evident in any physical demand variables between games that were won and lost in male players. Regarding comparisons according to competition phase, male players completed more HIA per minute (*P* = 0.014; *small* ES) and spent a greater proportion of playing time performing HIA during group games than finals games (*P* = 0.018; *small* ES) but spent a greater proportion of playing time performing REC during finals games than group games (*P* = 0.012; *small* ES). When considering singular high-intensity activities, male players performed more high-SM per minute and spent a greater proportion of playing time performing high-SM during groups compared to finals games (*P* < 0.001; *moderate* ES) but spent a higher proportion of playing time jumping during finals games compared to group games (*P* = 0.009; *small* ES). No significant interactions between fixed effects were evident for physical demand variables in male players (*P* > 0.05).

**TABLE 2:** Physical demands (mean ± standard deviation) according to game outcome (i.e., group games vs. finals games [quarter-finals, semi-finals, and finals]) during the male 2019 FIBA 3 × 3 World Cup.

Dependent variables	Fixed effect			CV%	*P* value	Estimate	95% CI for ES estimate		ES	95% CI for ES		Interpretation
							Lower	Upper		Lower	Upper	
Frequency of occurrence (n/min)												

REC	Game outcome	Win	5.29 ± 1.75	33.08	0.156	-0.37	-0.88	0.14	0.25	-0.14	0.64	*Small*
		Loss	5.73 ± 1.83	31.94								

	Phase	Group	5.36 ± 1.91	35.63	0.213	-0.34	-0.86	0.19	0.2	-0.19	0.6	*Small*
		Finals	5.74 ± 1.59	27.70								

LIA	Game outcome	Win	14.71 ± 1.24	8.42	0.247	0.28	-0.19	0.76	-0.25	-0.64	0.14	*Small*
		Loss	14.33 ± 1.76	12.28								

	Phase	Group	14.46 ± 1.58	10.93	0.722	-0.09	-0.57	0.40	0.10	-0.30	0.50	*Trivial*
		Finals	14.61 ± 1.47	10.06								

MIA	Game outcome	Win	8.00 ± 1.55	19.38	0.338	-0.27	-0.82	0.28	0.14	-0.25	0.52	*Trivial*
		Loss	8.22 ± 1.70	20.68								

	Phase	Group	8.31 ± 1.57	18.89	0.282	0.31	-0.25	0.86	-0.32	-0.72	0.08	*Small*
		Finals	7.79 ± 1.68	21.57								

HIA	Game outcome	Win	11.47 ± 2.13	18.57	0.249	0.46	-0.31	1.22	-0.17	-0.55	0.22	*Trivial*
		Loss	11.10 ± 2.20	19.81								

	Phase	Group	11.73 ± 1.97	16.79	**0.014**	0.99	0.22	1.76	-0.56	-0.97	-0.14	*Small*
		Finals	10.56 ± 2.29	21.69								

Total	Game outcome	Win	39.46 ± 2.59	6.56	0.743	0.17	-0.85	1.19	-0.03	-0.41	0.36	*Trivial*
		Loss	39.38 ± 3.26	8.28								

	Phase	Group	39.87 ± 2.73	6.85	0.129	0.8	-0.22	1.83	-0.41	-0.80	0.00	*Small*
		Finals	38.70 ± 3.12	8.06								

**Total time (%)**												

REC	Game outcome	Win	15.24 ± 7.13	46.78	0.115	-1.65	-3.68	0.38	0.21	-0.18	0.59	*Small*
		Loss	16.73 ± 7.29	43.57								

	Phase	Group	14.59 ± 5.97	40.92	**0.012**	-2.73	-4.81	-0.66	0.51	0.10	0.92	*Small*
		Finals	18.20 ± 8.46	46.48								

LIA	Game outcome	Win	44.41 ± 4.77	10.74	0.182	1.19	-0.54	2.93	-0.34	-0.73	0.05	*Small*
		Loss	42.66 ± 5.52	12.94								

	Phase	Group	43.57 ± 5.19	11.91	0.592	0.48	-1.28	2.24	-0.02	-0.41	0.38	*Trivial*
		Finals	43.48 ± 5.30	12.19								

MIA	Game outcome	Win	16.74 ± 3.40	20.31	0.572	-0.35	-1.54	0.85	0.15	-0.24	0.53	*Trivial*
		Loss	17.28 ± 3.81	22.05								

	Phase	Group	17.41 ± 3.50	20.10	0.446	0.47	-0.74	1.68	-0.29	-0.69	0.11	*Small*
		Finals	16.37 ± 3.72	22.72								

HIA	Game outcome	Win	23.61 ± 4.53	19.19	0.496	0.59	-1.1	2.28	-0.06	-0.44	0.33	*Trivial*
		Loss	23.34 ± 4.99	21.38								

	Phase	Group	24.43 ± 4.41	18.05	**0.018**	2.09	0.39	3.79	-0.54	-0.95	-0.12	*Small*
		Finals	21.95 ± 4.92	22.41								

*Abbreviations:* CV%, coefficient of variation as a percentage; CI, confidence intervals; ES, effect size; REC, recovery; LIA, low-intensity activities; MIA, medium-intensity activities; HIA, High-intensity activities; SM, specific movements. *Notes:* Bolded *P* value indicates statistically significant difference (*P* < 0.05).

**TABLE 3:** High-intensity activities (mean ± standard deviation) according to game outcome (i.e., win vs. loss) and competition phase (i.e., group games vs. finals games [quarter-finals, semi-finals, and finals]) during the male 2019 FIBA 3 × 3 World Cup.

Dependent variables	Fixed effect			CV%	*P* value	Estimate	95% CI for ES estimate		ES	95% CI for ES		Interpretation
							Lower	Upper		Lower	Upper	
**Frequency of occurrence (n/min)**												

Sprint	Game outcome	Win	1.94 ± 0.79	40.72	0.581	0.08	-0.19	0.35	-0.15	-0.53	0.24	*Trivial*
		Loss	1.83 ± 0.70	38.25								

	Phase	Group	1.93 ± 0.86	44.56	0.404	0.12	-0.16	0.39	-0.17	-0.57	0.22	*Trivial*
		Finals	1.80 ± 0.72	40.00								

High-SM	Game outcome	Win	6.34 ± 1.42	22.40	0.239	0.28	-0.18	0.75	-0.17	-0.56	0.21	*Trivial*
		Loss	6.11 ± 1.29	21.11								

	Phase	Group	6.61 ± 1.19	18.00	**< 0.001**	0.87	0.41	1.34	-0.78	-1.21	-0.35	*Moderate*
		Finals	5.62 ± 1.38	24.56								

Jump	Game outcome	Win	3.18 ± 0.84	26.42	0.462	0.12	-0.20	0.45	-0.02	-0.41	0.36	*Trivial*
		Loss	3.16 ± 0.99	31.33								

	Phase	Group	3.19 ± 0.92	28.84	0.905	-0.02	-0.35	0.31	-0.06	-0.45	0.34	*Trivial*
		Finals	3.14 ± 0.91	28.98								

**Total time (%)**												

Sprint	Game outcome	Win	2.74 ± 1.18	43.07	0.504	0.14	-0.26	0.54	-0.16	-0.55	0.22	*Trivial*
		Loss	2.56 ± 1.01	39.45								

	Phase	Group	2.68 ± 1.14	42.54	0.670	0.09	-0.31	0.49	-0.08	-0.47	0.32	*Trivial*
		Finals	2.59 ± 1.04	40.15								

High-SM	Game outcome	Win	17.03 ± 4.12	24.19	0.779	0.21	-1.25	1.67	0.02	-0.37	0.40	*Trivial*
		Loss	17.10 ± 4.38	25.61								

	Phase	Group	18.17 ± 3.93	21.63	**< 0.001**	2.56	1.09	4.03	-0.72	-1.14	-0.29	*Moderate*
		Finals	15.30 ± 4.14	27.06								

Jump	Game outcome	Win	3.85 ± 1.05	27.27	0.270	0.22	-0.17	0.61	-0.15	-0.53	0.24	*Trivial*
		Loss	3.68 ± 1.19	32.34								

	Phase	Group	3.58 ± 1.06	29.61	**0.009**	-0.53	-0.92	-0.14	0.44	0.03	0.84	*Small*
		Finals	4.06 ± 1.17	28.82								

*Abbreviations:* CV%, coefficient of variation as a percentage; CI, confidence intervals; ES, effect size; SM, specific movements. *Notes:* Bolded *P* value indicates statistically significant difference (*P* < 0.05).

Descriptive data and statistical analyses for physical demand variables during female basketball games according to game outcome (i.e., win vs. loss) and competition phase (i.e., group games vs. finals games) are presented in Tables 4 and 5. Regarding comparisons according to game outcome, no significant differences were evident in any physical demand variables between games that were won and lost in female players. Regarding comparisons according to competition phase, female players performed more LIA per minute (*P* = 0.018; *small* ES) and spent a greater proportion of playing time performing LIA (*P* = 0.010; *small* ES) in group games than finals games. No significant interactions between fixed effects were evident for physical demand variables in female players (*P* > 0.05).

**TABLE 4:** Physical demands (mean ± standard deviation) according to game outcome (i.e., win vs. loss) and competition phase (i.e., group games vs. finals games [quarter-finals, semi-finals, and finals]) during the female 2019 FIBA 3 × 3 World Cup.

Dependent variables	Fixed effect			CV%	*P* value	Estimate	95% CI for ES estimate		ES	95% CI for ES		Interpretation
							Lower	Upper		Lower	Upper	
**Frequency of occurrence (n/min)**												

REC	Game outcome	Win	4.96 ± 1.59	32.06	0.237	0.28	-0.18	0.73	-0.08	-0.45	0.29	*Trivial*
		Loss	4.83 ± 1.67	34.58								

	Phase	Group	5.03 ± 1.61	32.01	0.310	-0.23	-0.68	0.22	-0.19	-0.56	0.19	*Trivial*
		Finals	4.72 ± 1.65	34.96								

LIA	Game outcome	Win	15.20 ± 1.74	11.45	0.407	0.24	-0.32	0.80	-0.02	-0.39	0.35	*Trivial*
		Loss	15.17 ± 1.53	10.09								

	Phase	Group	15.50 ± 1.58	10.19	**0.018**	0.70	0.13	1.27	-0.47	-0.85	-0.08	*Small*
		Finals	14.76 ± 1.61	10.91								

MIA	Game outcome	Win	8.08 ± 1.58	19.55	0.609	-0.14	-0.65	0.38	0.18	-0.20	0.55	*Trivial*
		Loss	8.35 ± 1.41	16.89								

	Phase	Group	8.04 ± 1.40	17.41	0.290	-0.28	-0.80	0.24	0.27	-0.11	0.65	*Small*
		Finals	8.45 ± 1.59	18.82								

HIA	Game outcome	Win	11.95 ± 2.57	21.51	0.464	-0.31	-1.13	0.51	0.05	-0.32	0.42	*Trivial*
		Loss	12.08 ± 2.27	18.79								

	Phase	Group	11.75 ± 2.59	22.04	0.476	-0.30	-1.14	0.53	0.26	-0.12	0.64	*Small*
		Finals	12.37 ± 2.12	17.14								

Total	Game outcome	Win	40.20 ± 3.43	8.53	0.952	-0.04	-1.22	1.15	0.06	-0.31	0.43	*Trivial*
		Loss	40.42 ± 3.41	8.44								

	Phase	Group	40.32 ± 3.50	8.68	0.827	0.13	-1.06	1.33	-0.01	-0.38	0.37	*Trivial*
		Finals	40.30 ± 3.31	8.21								

**Total time (%)**												

REC	Game outcome	Win	14.11 ± 6.38	45.22	0.170	1.29	-0.54	3.11	-0.12	-0.49	0.25	*Trivial*
		Loss	13.34 ± 6.51	48.80								

	Phase	Group	13.90 ± 6.30	45.32	0.108	-1.50	-3.31	0.31	-0.06	-0.44	0.31	*Trivial*
		Finals	13.48 ± 6.66	49.41								

LIA	Game outcome	Win	44.89 ± 5.87	13.08	0.707	0.36	-1.50	2.22	-0.08	-0.45	0.29	*Trivial*
		Loss	44.41 ± 5.76	12.97								

	Phase	Group	45.40 ± 6.02	13.26	**0.010**	2.52	0.65	4.40	-0.30	-0.68	0.08	*Small*
		Finals	43.66 ± 5.38	12.32								

MIA	Game outcome	Win	16.70 ± 2.84	17.01	0.651	-0.23	-1.25	0.78	0.16	-0.21	0.53	*Trivial*
		Loss	17.15 ± 2.90	16.91								

	Phase	Group	16.54 ± 2.86	17.29	0.192	-0.68	-1.71	0.34	0.31	-0.07	0.69	*Small*
		Finals	17.43 ± 2.82	16.18								

HIA	Game outcome	Win	24.30 ± 4.82	19.84	0.166	-1.11	-2.66	0.45	0.17	-0.20	0.54	*Trivial*
		Loss	25.10 ± 4.65	18.53								

	Phase	Group	24.16 ± 4.79	19.83	0.214	-1.00	-2.57	0.57	0.27	-0.11	0.65	*Small*
		Finals	25.43 ± 4.60	18.09								

*Abbreviations*: CV%, coefficient of variation as a percentage; CI, confidence intervals; ES, effect size; REC, recovery; LIA, low-intensity activities; MIA, medium-intensity activities; HIA, High-intensity activities; SM, specific movements. *Notes:* Bolded *P* value indicates statistically significant difference (*P* < 0.05).

**TABLE 5:** High-intensity activities (mean ± standard deviation) according to game outcome (i.e., win vs. loss) and competition phase (i.e., group games vs. finals games [quarter-finals, semi-finals, and finals]) during the female 2019 FIBA 3 × 3 World Cup.

Dependent variables	Fixed effect			CV%	*P* value	Estimate	95% CI for ES estimate		ES	95% CI for ES		Interpretation
							Lower	Upper		Lower	Upper	
**Frequency of occurrence (n/min)**												

Sprint	Game outcome	Win	1.96 ± 0.97	49.49	0.098	-0.28	-0.62	0.05	0.23	-0.15	0.60	*Small*
		Loss	2.20 ± 1.10	50.00								

	Phase	Group	1.94 ± 0.93	47.94	0.251	-0.20	-0.53	0.14	0.32	-0.06	0.70	*Small*
		Finals	2.27 ± 1.15	50.66								

High-SM	Game outcome	Win	6.57 ± 1.43	21.77	0.208	-0.30	-0.75	0.16	0.11	-0.26	0.48	*Trivial*
		Loss	6.72 ± 1.31	19.49								

	Phase	Group	6.45 ± 1.45	22.48	0.230	-0.28	-0.75	0.18	0.33	-0.06	0.70	*Small*
		Finals	6.89 ± 1.23	17.85								

Jump	Game outcome	Win	3.43 ± 1.00	29.15	0.148	0.25	-0.09	0.59	-0.28	-0.66	0.09	*Small*
		Loss	3.17 ± 0.85	26.81								

	Phase	Group	3.36 ± 1.00	29.76	0.291	0.19	-0.16	0.53	-0.16	-0.53	0.22	*Trivial*
		Finals	3.21 ± 0.85	26.48								

**Total time (%)**												

Sprint	Game outcome	Win	2.88 ± 1.43	49.65	0.061	-0.53	-1.08	0.02	0.30	-0.07	0.68	*Small*
		Loss	3.39 ± 1.84	54.28								

	Phase	Group	2.94 ± 1.53	52.04	0.368	-0.26	-0.81	0.30	0.27	-0.11	0.65	*Small*
		Finals	3.39 ± 1.80	53.10								

High-SM	Game outcome	Win	17.97 ± 3.86	21.48	0.165	-0.91	-2.19	0.37	0.17	-0.20	0.54	*Trivial*
		Loss	18.63 ± 3.81	20.45								

	Phase	Group	17.87 ± 3.89	21.77	0.123	-1.03	-2.32	0.27	0.26	-0.12	0.64	*Small*
		Finals	18.87 ± 3.72	19.71								

Jump	Game outcome	Win	3.45 ± 1.07	31.01	0.063	0.34	-0.01	0.69	-0.37	-0.74	0.01	*Small*
		Loss	3.09 ± 0.85	27.51								

	Phase	Group	3.35 ± 1.02	30.45	0.213	0.23	-0.13	0.58	-0.18	-0.56	0.19	*Trivial*
		Finals	3.17 ± 0.91	28.71								

*Abbreviations:* CV%, coefficient of variation as a percentage; CI, confidence intervals; ES, effect size; SM, specific movements. *Notes:* Bolded *P* value indicates statistically significant difference (*P* < 0.05).

## DISCUSSION

This study is the first to compare the physical demands encountered during 3 × 3 basketball games according to game outcome and competition phase. Furthermore, this study explored player demands in both male and female competitions during the World Cup, the highest international competition standard in 3 × 3 basketball. In turn, findings demonstrated no significant differences in physical demands between games that were won and lost in both male and female competitions. Comparisons in demands between competition phases revealed greater HIA bouts per minute, specifically high-SM, and a lower proportion of playing time spent in recovery and executing jumps were performed during group games compared to finals games in male players. In contrast, greater LIA were performed during group games compared to finals games in female players.

The similar demands between games that were won and lost during the 2019 FIBA 3 × 3 World Cup suggest that the activity outputs of players were homogenous across opposing teams and did not exert a dominant effect on dictating game results. Reasons for these findings may be related to the configurations of 3 × 3 basketball games limiting the extent to which differences in the physical capabilities of players manifest into the activity completed during games. Specifically, the short playing time (i.e., maximum 10 min) and small playing area (15 m × 11 m) likely restricts the activity outputs able to be generated (e.g., no transitions up and down a full court) and limits the effects of activity-induced fatigue during 3 × 3 basketball games [7], keeping activity demands relatively stable across teams. Furthermore, separate studies demonstrated players competing at the 2016 FIBA 3 × 3 World Championships possessed greater physical capabilities via clear differences in various fitness tests (males: aerobic capacity measured via the Yo-Yo Intermittent Recovery Test Level 1; females: change-of-direction speed via the Agility 505 Test) than players competing at the 2016 Under 18 World Championships [25], yet produced similar activity outputs during games measured using PlayerLoad · min^−1^ [8].

Comparisons to past research are difficult given the lack of research exploring physical demands according to game outcome in 3 × 3 basketball; however, findings from 5 × 5 basketball show similar trends. Specifically, previous data indicate semi-professional, male basketball players perform similar (*unclear* to *small* effects) external loads during games that were won and lost (PlayerLoad and PlayerLoad · min^−1^) [17] and during quarters that were won and lost (peak PlayerLoad · min^−1^ across 15-s to 5-min sample durations and average PlayerLoad · min^−1^) [17]. Nevertheless, while physical demands have not been quantified according to game outcome in 3 × 3 basketball, research has compared technical-tactical variables between winning and losing teams during 3 × 3 basketball games. Specifically, winning male and female teams competing in the 2019 3 × 3 European Basketball Cup performed scoring more efficiently, secured more rebounds, committed fewer turnovers, and committed less fouls than losing teams [3]. Indeed, similar findings have been consistently reported in 5 × 5 basketball competitions, with various technical-tactical variables significantly favouring winning compared to losing teams in the Euroleague (i.e., assists, blocked shots, fouls, turnovers, shots made, and shooting percentage) [26], Australian National Basketball League (i.e., shots made, defensive rebounds, blocked shots, assists, steals, fouls, and turnovers) [27], and FIBA Asian and European Women’s Championships (i.e., shots made, rebounds, blocked shots, assists, steals, and turnovers) [28]. Consequently, past findings combined with those in this study indicate that tactical and/or skill differences might differentiate team success more than physical outputs in high-level basketball games following 3 × 3 and 5 × 5 formats. In turn, technical-tactical elements may be particularly important to consider when developing and/or selecting players for 3 × 3 basketball competitions. However, given that existing technical-tactical data for 3 × 3 basketball games were indicative of the 2019 European Basketball Cup [3] and the physical data provided in this study were indicative of the 2019 World Cup, further research exploring technical-tactical and physical demands collectively in the same 3 × 3 basketball competition are encouraged to confirm this notion.

Unlike game outcome, significant differences emerged in the physical demands encountered during group games compared to finals games. More precisely, in the male competition at the 2019 World Cup, the lower HIA and high-SM bouts per minute and greater proportion of playing time spent in recovery and jumping in finals games compared to the group phase might be attributed to an increased defensive pressure being applied in finals games. In this regard, teams may have adopted tighter defensive structures during finals games compared to group games, limiting the freedom of opponents to move at high intensities as well as contested shots more effectively resulting in more jumping activity (i.e., attempting to block shots, shooting over defender’s hands, and securing rebounds). In contrast to male players, female players at the 2019 3 × 3 World Cup performed significantly less LIA during finals games compared to group games, suggesting increased activity was dispersed among moderate and high intensities to a greater extent in finals games. This trend in activity for female players may be expected given the increased opponent quality faced in finals games may evoke higher activity demands and/or players may place increased importance on finals games given the proximity to deciding the competition outcome [29] and consequently work at greater activities intensities than during group games. Nevertheless, it should be noted that the significant differences in physical demands in male and female players between group and final games were *small* in magnitude, with many variables not reaching significance. In this regard, past research examining a large sample of male (n = 250) and female (n = 201) basketball players competing in the 2016 FIBA 3 × 3 under-18 World Championships, Senior European and World Championships, and selected FIBA World Tour events showed no clear change in external load (i.e., Player-Load · min^1^) or internal load (i.e., blood lactate concentration and average heart rate) variables from the first group game to the championship game [7]. Likewise, few technical-tactical demand variables were shown to differ between group and finals games (i.e., higher points per possession and less blocked shots during finals) in male and female teams competing in the 2019 3 × 3 European Basketball Cup [3]. Consequently, the collective 3 × 3 basketball research suggests the physiological, physical, and technical-tactical demands are rather consistent across competitions phases. Thus, the present findings should not be overstated as it appears that competition phase may only partially impact (i.e., have *small* effects) the physical demands experienced by players in the context of the 2019 3 × 3 World Cup.

The novel findings presented in this study should be interpreted while considering the associated limitations. First, male and female competitions in the 2019 3 × 3 World Cup were examined in this study, so the reported data may not be indicative of other 3 × 3 competitions. Second, physical demand variables were determined using video-based TMA with subjective tester interpretation of video footage, so further research complementing these analyses with objective approaches to quantify the physical demands (e.g., local positioning system or inertial sensor technologies) and internal stress imposed on players according to each contextual factor are encouraged to provide a more holistic indication of the demands encountered during 3 × 3 basketball games. Third, key contextual factors in the form of game outcome and competition phase were selected for analyses in this study given they have been examined in previous basketball research [17, 18, 30] to provide useful insight for endusers. However, other contextual factors shown to impact the physical demands placed on basketball players during games such as opponent quality [31], game location [17], and score-line [17] warrant consideration in future research. Fourth, the video sourced online had a small portion of footage missing (< 3%) for some games; however, it is anticipated that due to the brief footage missed for a selection of games, the overall physical demands reported were not adversely impacted. Finally, games were removed from analyses where lower-ranked teams (i.e., teams that did not make the finals phase) played one another to permit more even comparisons between group and finals phases in the present study. Consequently, the comparative analyses presented in this study are indicative of better-ranked teams who reached the finals phase at the 2019 3 × 3 World Cup.

### Practical applications

The present findings hold useful practical applications in various ways. The similar physical demands experienced in games that were won and lost suggest team success may not be predicated on physical capabilities in international 3 × 3 basketball competition. In turn, players competing at the highest standard of play (i.e., World Cup) may possess rather homogenous physical capacities given the elite level of competition. Nevertheless, it is essential that the physical preparation of players is given adequate consideration alongside technical-tactical skills through appropriate training and recovery plans to ensure players reach the requisite level of fitness needed to compete in 3 × 3 international basketball competition. In this regard, it appears that the reduced playing area associated with 3 × 3 compared to 5 × 5 basketball limits the ability of players to perform sprints on a repeated basis, with most high-intensity activity captured in the present study consisting of basketball-specific movements (i.e., stance position, shuffling, rolling, reversing, screening, and cross-over running). Consequently, our data support previous recommendations advocating the need to develop acceleration, deceleration, and change-of-direction attributes through appropriate strength and conditioning approaches in 3 × 3 basketball given the reduced running demands compared to traditional 5 × 5 basketball [8]. Furthermore, our data emphasise 3 × 3 basketball players require well-developed vertical power expression with > 3 jumps performed per minute on average during games, including significantly greater playing time spent jumping in finals games compared to group games in male players. Despite some variations in physical demands between group and finals games, most variables were consistent between competition phases similar to external and internal load data reported in other international 3 × 3 basketball competitions [7]. These data suggest players may have adequate fitness capacities and/or adopt effective recovery strategies between games to offset an accumulative fatigue and maintain activity outputs across competition phases in light of the short game durations and tournament setting during the 2019 3 × 3 World Cup.

## CONCLUSIONS

The present findings add to the limited knowledge surrounding the physical demands encountered during 3 × 3 basketball game-play, providing foundation data specifically during the 3 × 3 World Cup. In turn, physical player demands were consistent irrespective of whether teams won or lost in both male and female competitions. Furthermore, although physical player demands differed for some variables during finals games compared to group games in male players (HIA and high-SM decreased while jumping duration decreased in finals) and female players (LIA decreased in finals), these effects were mostly *small* in magnitude with many variables remaining consistent between competition phases.
